# Prognostic value and immune landscapes of TERT promoter methylation in triple negative breast cancer

**DOI:** 10.3389/fimmu.2023.1218987

**Published:** 2023-07-28

**Authors:** Fei Lin, Jiajia Huang, Wancui Zhu, Tongchao Jiang, Jia Guo, Wen Xia, Miao Chen, Ling Guo, Wuguo Deng, Huanxin Lin

**Affiliations:** State Key Laboratory of Oncology in South China, Sun Yat-sen University Cancer Center, Guangzhou, China

**Keywords:** triple negative breast cancer (TNBC), telomerase reverse transcriptase (TERT), methylation, prognostic value, immune infiltration

## Abstract

**Background:**

Treatment options for patients with triple-negative breast cancer (TNBC) remain limited to mainstay therapies owing to a lack of efficacious therapeutic targets. Accordingly, there is an urgent need to discover and identify novel molecular targets for the treatment and diagnosis of this disease. In this study, we analyzed the correlation of telomerase reverse transcriptase (TERT) methylation status with TERT expression, prognosis, and immune infiltration in TNBC and identified the role of TERT methylation in the regulation TNBC prognosis and immunotherapy.

**Methods:**

Data relating to the transcriptome, clinicopathological characteristics and methylation of TNBC patients were obtained from The Cancer Genome Atlas (TCGA) database. TERT expression levels and differential methylation sites (DMSs) were detected. The correlations between TERT expression and DMSs were calculated. Kaplan–Meier curves was plotted to analyze the relationship between the survival of TNBC patients and the DMSs. The correlations of DMSs and TERT expression with several immunological characteristics of immune microenvironment (immune cell infiltration, immunomodulators, immune-related biological pathways, and immune checkpoints) were assessed. The results were validated using 40 TNBC patients from Sun Yat-sen University Cancer Center (SYSUCC).

**Results:**

Six DMSs were identified. Among them, four sites (cg11625005, cg07380026, cg17166338, and cg26006951) were within the TERT promoter, in which two sites (cg07380026 and cg26006951) were significantly related to the prognosis of patients with TNBC. Further validation using 40 TNBC samples from SYSUCC showed that the high methylation of the cg26006951 CpG site was associated with poor survival prognosis (*P*=0.0022). TERT expression was significantly correlated with pathological N stage and clinical stage, and cg07380026 were significantly associated with pathological T and N stages in the TCGA cohort. Moreover, the methylation site cg26006951, cg07380026 and TERT expression were significantly correlated with immune cell infiltration, common immunomodulators, and the level of the immune checkpoint receptor lymphocyte activation gene 3 (LAG-3) in TNBC patients.

**Conclusion:**

TERT promotertypermethylation plays an important role in TERT expression regulation and tumor microenvironment in TNBC. It is associated with overall survival and LAG-3 expression. TERT promoter hypermethylation may be a potential molecular biomarker for predicting response to the TERT inhibitors and immune checkpoint inhibitors in TNBC.

## Introduction

Breast cancer is the most common malignant tumor in women according to the latest epidemiological data from 2022 (1). Breast cancer is classified into different subtypes based on estrogen receptor (ER), progesterone receptor (PR), and human epidermal growth factor receptor 2 (HER-2) expression (2). Chemotherapy, radiotherapy, endocrine therapy, and targeted therapy are all appropriate postoperative treatment options for each subtype (3, 4). Triple-negative breast cancer (TNBC) constitutes a breast cancer subtype with obvious heterogeneity regarding histomorphology, genetic background, treatment response, and prognosis (5, 6). Chemotherapy remains the first-choice treatment for TNBC owing to a lack of other, more effective treatment methods as well as the aggressive biological behavior of this cancer (7), which is associated with a high risk of early recurrence, particularly visceral metastasis (8, 9). However, over recent years, immunotherapy has brought hope for patients with TNBC (10). Current research hotspots involving the immune microenvironment in TNBC include PD-L1 expression, evaluation of tumor-infiltrating lymphocytes (TILs) (11), and distribution of immune cell subsets, among others (USCAP, 2022) (12). The biggest advantage of immunotherapy lies in that the body’s own immune system is used to attack the tumor cells (13). Additionally, the tumor cells are specifically recognized by the immune system (adaptive/acquired immunity), thus potentially avoiding the targeting of non-cancerous cells. Moreover, immunological memory results in that once tumor-specific memory lymphocytes are generated, they will exist for life, and will play a role in tumor eradication after recurrence or metastasis. Finally, immunotherapy has relatively weak cytotoxicity. Because TNBC is associated with a high mutation rate, TIL ratio, and PD-L1 expression, patients with this type of breast cancer represent a population that may potentially benefit from immunotherapy (14).

The telomerase reverse transcriptase (TERT) gene, which encodes a catalytic subunit of the telomerase enzyme, plays an important role in tumorigenesis by maintaining telomere homeostasis and the proliferative capacity of cells (15, 16). TERT is located on the short arm of human chromosome 5 (5p15.33) and is 42 kb long, including 15 introns, 16 exons, and a 330-bp promoter region (17). Most human somatic cells do not possess telomerase activity as TERT transcription is suppressed during embryonic development (18, 19). However, TERT expression and telomerase activity are reactivated during tumorigenesis and tumor progression and are considered to be biomarkers of tumor cells (20, 21). Telomerase acts to maintain the length and stability of telomeres, as well as the ability of cells to proliferate indefinitely, as observed in cancer cells (22, 23). Recent studies have shown that, in addition to maintaining telomere length, TERT is also involved in multiple signaling pathway, such as those associated with the MAPK and PI3K/Akt/mTOR signaling pathway, the WNT/β-catenin signaling pathway, the NF-κB signaling pathway, and epithelial-mesenchymal transition-related pathways (24–27).

TERT has been reported to function as an oncogene in various types of cancer (28–31). Many factors contribute to the abnormal elevation of TERT expression levels, including transcriptional activators, TERT copy number variation, TERT promoter mutation, and TERT hypermethylation, among others (32). TERT is mutated in many solid tumors, including central nervous system malignancies, thyroid cancer, and melanoma (33–36). The two most common mutated sites are 1,295,228 C>T and 1,295,250 C>T (C228T and C250T, respectively) (37, 38). Mutations mentioned above, which result in changes in transcription factor binding sequences, enhance TERT transcriptional activity, which is one of the main mechanisms underlying the upregulation of TERT expression. But TERT promoter hypermethylation is more prevalent (>70%) in cancer types without TERT mutation (e.g., lung, breast, prostate, and colon cancers) (18, 39), implying that epigenetic mechanisms are the drivers of upregulation of TERT expression in these cancer types (40, 41). However, the exact locations of the epigenetic modifications and their effect on TERT promoter activation remain unclear. A methylation map based on next-generation sequencing showed that DNA methylation was not uniform along the TERT promoter region (18).

To further clarify the specific situation of TERT promoter methylation and its clinical significance, in this study, we explored the association of the CpG sites in the TERT promoter and TERT expression with clinicopathological characteristics, immunological features, and survival outcome in patients with TNBC.

## Methods

### Data retrieval

#### The Cancer Genome Atlas (TCGA) cohort

Data relating to the transcriptome, clinicopathological characteristics, and methylationome for the BRCA cohort were downloaded from TCGA database (https://portal.gdc.cancer.gov/). TNBC-related data were extracted from the whole BRCA cohort.

The Infinium HumanMethylation450 BeadChip methylation data were processed using the R package “ChAMP”. A gene expression matrix was constructed based on Fragments per Kilobase Million values. CpG sites in the human TERT (hTERT) gene were identified based on the methylation data of TNBC patients. Analysis of all CpG sites in hTERT between TNBC and normal adjacent tissues was conducted using the R package “edgeR” (version 3.34.1) (42, 43). In addition, the “pheatmap” (version 1.0.12) and “ggplot2” (version 3.3.5) packages were utilized to plot a heatmap and boxplot, respectively, for data visualization. The R package “DESeq2” (44) was employed to identify differences in TERT gene expression between TNBC samples and normal samples from TCGA database. The acquired data were visualized using a boxplot created using the “ggplot2” package (version 3.3.5). Correlations between the TERT gene and DMSs were calculated using the R package “ggstatsplot” (version 0.9.1). To analyze the relationship of DMSs with the survival of TNBC patients, Kaplan-Meier curves were constructed using the R package “survminer” (version 0.4.9).

#### Sun Yat-sen University Cancer Center (SYSUCC) cohort

For validation, a total of 40 patients were collected from SYSUCC, including 40 tumor tissue samples and 27 adjacent normal tissue samples (total 67 samples) from TNBC patients who underwent mastectomy or lumpectomy from December 2010 to November 2012.

### Methylation analysis

The methylation status of the cg26006951 and cg07380026 CpG sites was validated in the SYSUCC cohort using pyrosequencing. Briefly, DNA was extracted from clinical specimens using a TIAN amp Genomic DNA Kit (Cat. No: 4992254; ID: DP304-03, TIANGEN) and then subjected to bisulfite conversion following the manufacturer’s instructions. Purified bisulfite-converted DNA was amplified by PCR using primers covering the cg26006951 and cg07380026 CpG sites. The PCR products were subjected to pyrosequencing using the PyroMark Q96 pyrosequencing and quantification platform following the manufacturer’s instructions (Shanghai Biotechnology Corporation, Shanghai, China). The sequences of the primers used for amplification of the cg26006951 and cg07380026 target regions were forward: 5′-AGAAAGGGTGGGAAATGG-3′; reverse: 5′-ACCAAATATTAACCTCATCTACCA-3′ and forward: 5′-GAGAAAGGGTGGGAAATGGA-3′; reverse: 5′-ATAACCAAATATTAACCTCATCTACCA-3′, respectively.

### Antibodies, reagents, and immunohistochemistry

Slides were deparaffinized, rehydrated, immersed in an antigen-retrieval solution (pH 6), and boiled three times, 10 min each time, at a medium baking temperature in a microwave. After blocking with 3% BSA, the sections were incubated first with primary antibodies against TERT (dilution: 1:100; ID: ab32020, Company: Abcam; https://www.abcam.com/) and LAG-3 (dilution: 1:100; Cat No. 29548-1-AP, Company: Proteintech; https://www.ptglab.com/) at 4°C overnight in a humidified container and then with HRP-labeled anti-rabbit IgG secondary antibody. The specimens were counterstained with hematoxylin. The slides were scanned using a 3DHISTECH scanner (3DHISTECH Ltd, Budapest, Hungary). The immunostaining was evaluated independently by two pathologists blinded to the clinicopathologic information. The expression of TERT and LAG-3 (also known as CD223) was represented by the H-score. Antibody staining intensity was categorized as follows: no staining = 0, weak = 1, moderate = 2, and strong = 3. A five-scale system was used to categorize the percentage of stained cells, as follows: 0, no positive cells; 1, <25% positive cells; 2, 25%-50% positive cells; 3, 50%-75% positive cells; and 4, >75% positive cells. The H-score (range: 0-12) for each tissue was calculated by multiplying the intensity index with the percentage scale. The median value of the H-score for TERT staining served as the cutoff. Tumors with H-scores for TERT staining lower than or equal to the median were designated as “low expression”, whereas those with scores higher than the median were designated as “high expression”.

### Analysis of immune cell infiltration

The single-sample gene set enrichment analysis (ssGSEA) algorithm based on 24 types of immune cells was used to calculate immune cell infiltration in the tumor microenvironment in TNBC samples and normal samples from TCGA database. In addition, differences in immune cell infiltration between the TNBC and normal groups were analyzed using the rank-sum test (p<0.05) and visualized using the R package “ggplot2” (version 3.3.5). Subsequently, the package “ggstatsplot” (version 0.9.1) was employed to further analyze the relationships of the TERT gene and DMSs with 24 types of immune cells. Immune-related biological pathways in TNBC patients were further quantified using the R package “gsva” (version 1.40.1) (45).

### Analysis of correlations with immunomodulators

Spearman’s algorithm was used to analyze the correlations among TERT gene expression, TERT DMSs, and 74 immune modulators. The data were visualized in a heatmap.

### Follow-up

Follow-up was performed by telephone or through a regular outpatient surveillance system to record the condition of patients or the cause and date of death if the patient had already died. Follow-up duration was measured from the date of diagnosis to the date of the last visit or the date of death.

### Statistical analysis

Chi-square tests (categorical variables) or Mann–Whitney U tests (continuous variables) were used for comparisons between two groups. An optimal cut-off point for continuous variables was determined using maximally selected rank statistics. The significance of the Kaplan–Meier survival curve was assessed based on the log-rank test. Statistical analysis was conducted using GraphPad Prism (Version 8) and R (Version 4.0.3) software. Correlation coefficients were calculated using Spearman’s rank correlation. All p-values were from a two-tailed t-test and a p-value <0.05 was considered significant.

### Ethics statement

The clinical specimens used in this study were obtained from the Tumor Biobank of SYSUCC. The study protocol for the SYSUCC cohort was approved by the Institutional Research Ethics Committee of SYSUCC (B2022-332-01).

## Results

### TERT promoter was hypermethylated in tumor tissues compared with normal adjacent tissues in TNBC

**Figure 1:**
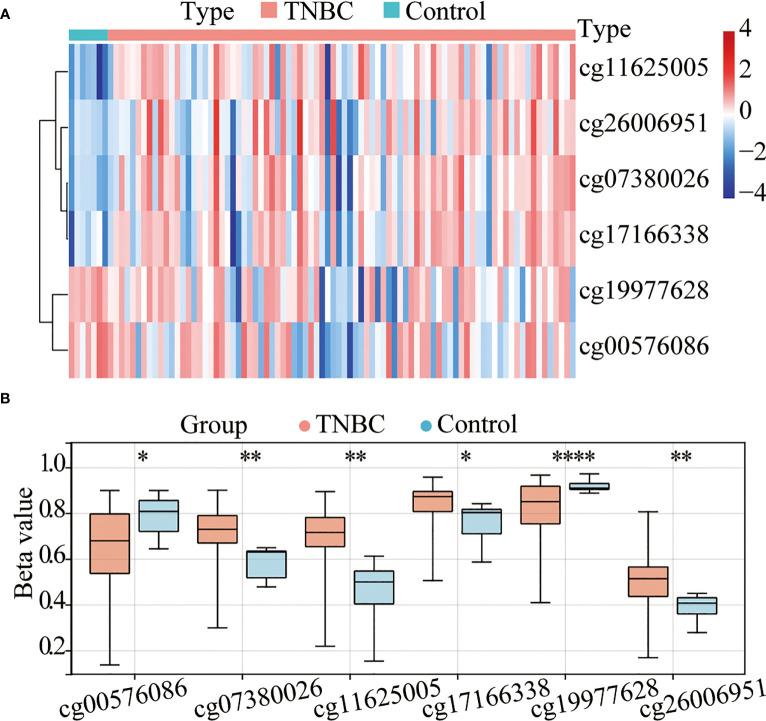
The TERT promoter is hypermethylated in triple-negative breast cancer (TNBC). **(A)** Heatmap of the differentially methylated CpG sites in TERT between tumor and adjacent normal tissues using data from The Cancer Genome Atlas (TCGA) cohort. **(B)** The boxplot of relative methylation levels of six differentially methylated CpG sites (cg00576086, cg07380026, cg11625005, cg17166338, cg19977628, and cg26006951) between tumor and adjacent normal tissues in the TCGA cohort. *P < 0.05; ** P < 0.01; **** P < 0.0001.

A total of 91 TNBC tissues in TCGA (84 tumor tissues and 7 normal tissues) with both methylation and expression data were selected for the combined methylation and transcriptome analysis. According to the annotation of the Infinium HumanMethylation450 BeadChip, 100 probes are located in TERT gene, 12 of which missing values. Accordingly, 88 valid TERT probes were used for detection (**Supplementary Table 1**). Using a β-value >0.4 and a p-value <0.05 as a cutoff, six CpG sites (**Figures 1A, B**) were found to be significantly differentially methylated, namely, cg11625005, cg07380026, cg17166338, cg26006951, cg19977628, and cg00576086. Among them, cg11625005, cg07380026, cg17166338, and cg26006951 were hypermethylated and cg19977628 and cg00576086 were hypomethylated. Moreover, these four sites (cg11625005, cg07380026, cg17166338, and cg26006951) were located in TERT TSS1500 or 5’UTR.

### TERT promoter methylation was positively correlated with TERT expression in patients with TNBC

**Figure 2:**
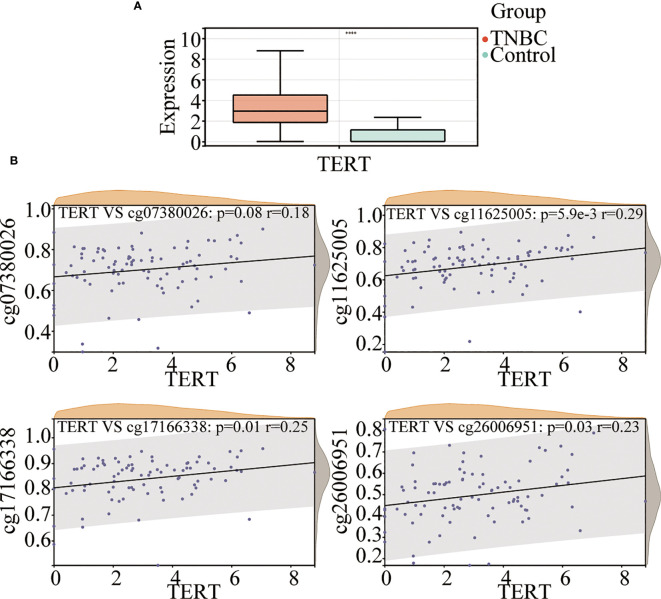
TERT promoter methylation is correlated with TERT expression in TNBC. **(A)** Comparison of TERT mRNA level between tumor and normal adjacent tissues in the TCGA cohort. **(B)** The correlation of cg07380026, cg11625005, cg17166338, and cg26006951 methylation status with TERT expression in tumor tissues in the TCGA cohort. **** P < 0.0001.

To investigate the role of TERT in TNBC, we analyzed TERT expression in both tumor tissues and normal tissues. The results showed that TERT mRNA expression was significantly higher in TNBC tissues than in normal tissues (**Figure 2A**). The methylation of gene promoter plays an important role in the regulation of expression. To investigate the effect of TERT promoter methylation on the regulation of TERT expression, we analyzed the correlation between the degree of methylation of the four CpG sites and TERT expression. We found that TERT expression was significantly and positively correlated with hypermethylation of cg11625005 (p < 0.0001, r = 0.29), cg17166338 (p = 0.01, r = 0.25), and cg26006951 (p = 0.03, r = 0.23) (**Figure 2B**).

### TERT promoter methylation predict overall survival in patients with TNBC

**Figure 3:**
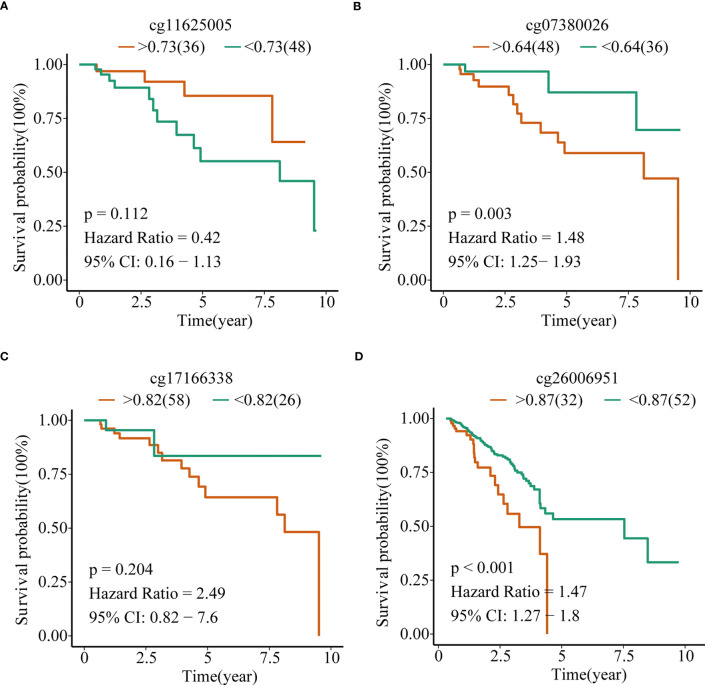
TERT promoter methylation predict overall survival in TNBC in the TCGA cohort. Kaplan–Meier analysis of overall survival relating to the methylation level of **(A)** cg11625005, **(B)** cg07380026, **(C)** cg17166338, and **(D)** cg26006951 in patients in the TCGA cohort, respectively.

To explore the prognostic value of TERT promoter methylation status in TNBC patients, we conducted Kaplan-Meier survival analyses relating to the four differentially methylated CpG sites with overall survival in TNBC patients from TCGA cohort. The results indicated that hypermethylation of the two sites (cg07380026 [p = 0.003, HR = 1.48, 95% CI: 1.25-1.93] and cg26006951 [p < 0.001, HR = 1.47, 95% CI: 1.27-1.80]) were associated with poor overall survival (**Figures 3A–D**).

**Figure 4:**
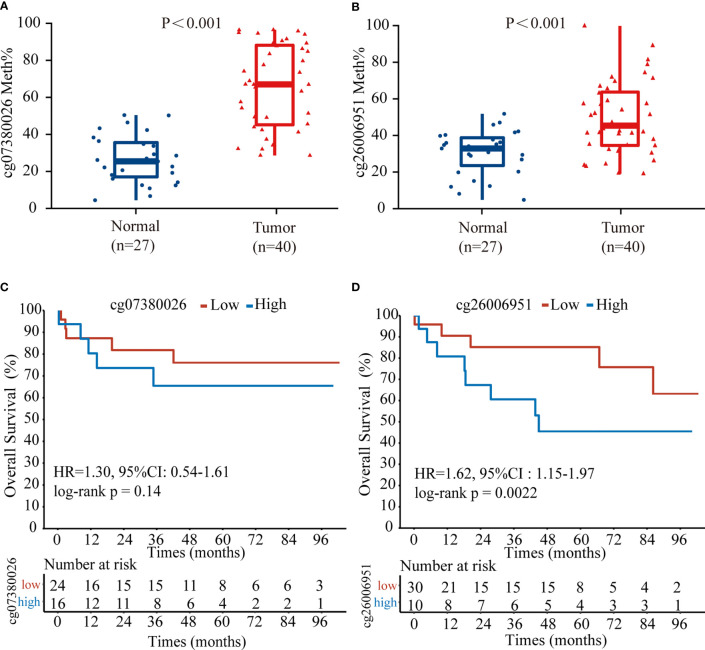
The CpG site cg26006951 predict overall survival in TNBC from Sun Yat-Sen University Cancer Center (SYSUCC) cohort. Comparison of the relative **(A)** cg07380026 and **(B)** cg26006951 methylation levels between tumor and normal adjacent tissues in the SYSUCC cohort. **(C, D)**. Kaplan–Meier survival analysis associated with cg07380026 and cg26006951 methylation level in the SYSUCC cohort, respectively.

To confirm the above results, we examined the methylation status of the cg26006951 and cg07380026 CpG sites in 40 TNBC samples from SYSUCC by pyrosequencing. The results showed that both cg26006951 and cg07380026 CpG sites were significantly hypermethylated in tumor tissues compared with that in normal adjacent tissues (**Figures 4A, B**) and hypermethylation of cg26006951 was associated with poor overall survival (cg07380026 [HR = 1.30, 95% CI: 0.54–1.61, log-rank p = 0.14] and cg26006951 [HR = 1.62, 95% CI: 1.15–1.97,log-rank p = 0.0022]) (**Figures 4C, D**).

Based on the above analysis, we showed that there is abnormal hypermethylation of the TERT promoter, and the hypermethylation of the CpG site cg26006951 is closely related to the poor prognosis in TNBC. Therefore, we speculate that the CpG site cg26006951 is a potential predictive biomarker for TNBC prognosis. Next, we focused on cg26006951 for further analysis. We divided the above 40 patients of SYSUCC into a cg26006951 hypermethylation group and a cg26006951 hypomethylation group according to the median value and analyzed the relationship between the clinicopathological characteristics and the methylation level of cg26006951 in TNBC patients. The results showed that there was no statistical correlation between the degree of methylation of cg26006951 and the patient’s age, T stage, N stage, clinical stage, and Ki-67 (**Table 1**).

**Table 1:** The relationship between cg26006951 and clinicopathologic characteristics in the SYSUCC cohort.

Characteristic	Total	cg26006951		*P*
	n=40	Low	High	
**Age (years) median (IQR)**	47 (39-56)	41 (36-46)	43 (37-48)	0.190
**Tumor stage**				0.216
T1	16 (40.0)	8 (50.0)	8 (50.0)	
T2	22 (55.0)	12 (54.5)	10 (45.5)	
T3	1 (2.5)	1 (100.0)	0 (0.0)	
T4	1 (2.5)	0 (0.0)	1 (100.0)	
**Node stage**				0.083
N0	22 (55.0)	11 (50.0)	11 (50.0)	
N1	11 (27.5)	4 (36.4)	7 (63.6)	
N2	0 (0.0)	0 (0.0)	0 (0.0)	
N3	7 (17.5)	6 (85.7)	1 (14.3)	
**Clinical stage**				0.078
I	8 (20.0)	4 (50.0)	4 (50.0)	
II	19 (47.5)	13 (68.4)	6 (31.6)	
III	13 (32.5)	8 (61.5)	5 (38.5)	
**Ki-67**				0.154
>14%	31 (77.5)	21 (67.7)	10 (32.3)	
≤14%	9 (22.5)	6 (66.7)	3 (33.3)	

According to the results of the TCGA database, we showed that cg07380026 and cg26006951 were potential poor prognostic factors for TNBC patients, and externally validated with 40 cases of TNBC tissues from the SYSUCC. Although the cg07380026 CpG site did not show statistical significance, the trend of the results was consistent with the TCGA database. Considering the existence of unavoidable interference factors such as small sample size, single-center samples and experimental errors, we believe that it is not possible to simply generalize from a statistical point of view and ignore its true clinical significance. Therefore, in the following further analysis of the TCGA database, we included TERT expression, cg07380026 and cg26006951 methylation levels into analysis.

### TERT gene expression and DMSs were closely related to the clinicopathological features of TNBC patients

**Figure 5:**
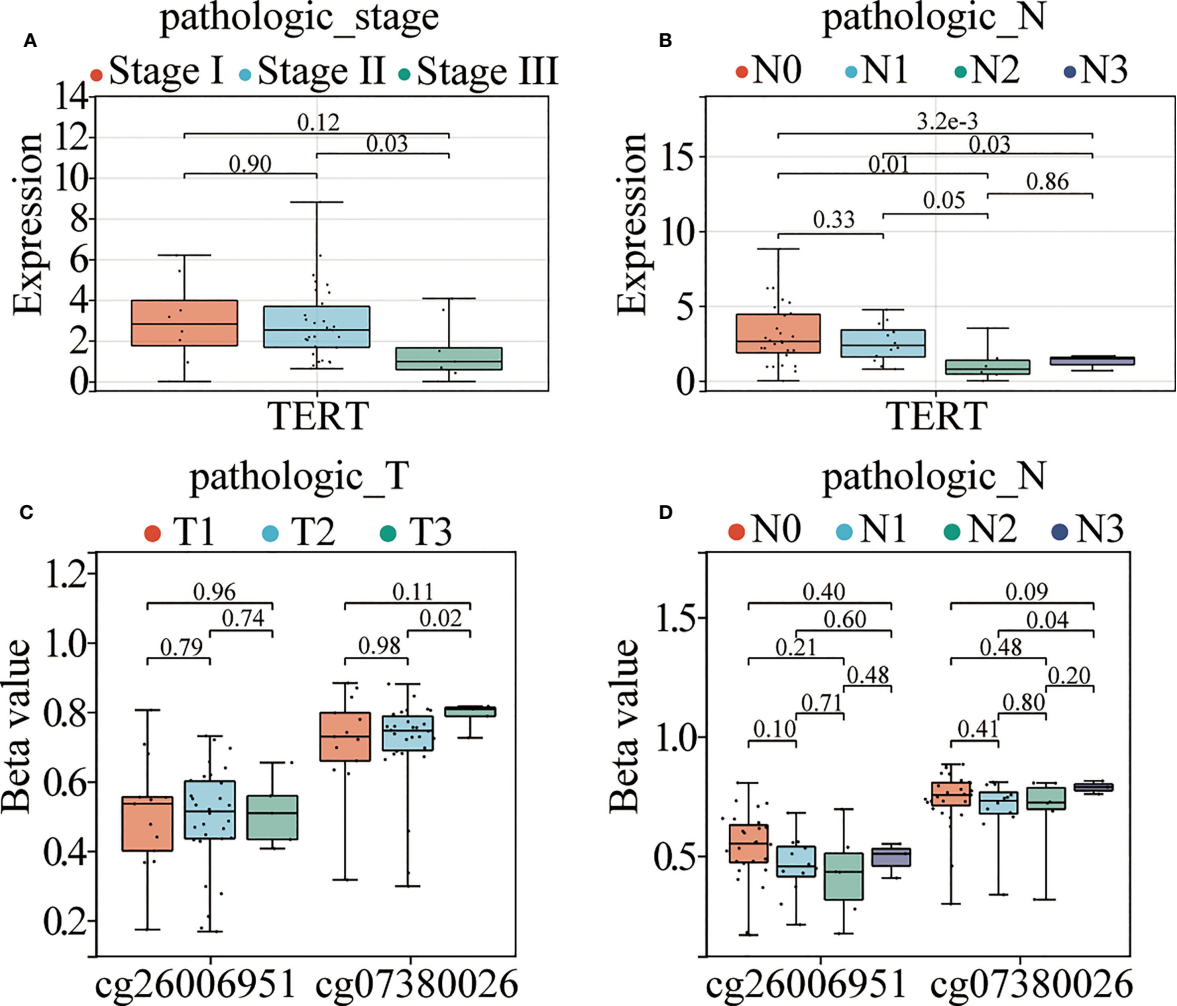
TERT promoter hypermethylation and elevated expression correlate with aggressive clinical phenotypes in TNBC. **(A**, **B)**. TERT expression in different TNM stages and pathological N stages among patients from the TCGA cohort. **(C, D)**. The relative methylation levels of cg26006951 and cg07380026 in different pathological N and T stages in patients in the TCGA cohort.

To further explore the association between TERT expression, cg07380026, cg26006951, and clinicopathological features of TNBC patients, we combined the information of age, T, N, M stage and clinical stage of TNBC patients of TCGA database (**Supplementary Table 2**) for correlation analysis. The results showed that TERT expression was significantly correlated with pathological N stages, specifically: N0 and N3 (p<0.0001), N0 and N2 (p=0.01), N1 and N2 (p=0.05) and N1 and N3 (p=0.03) and in clinical stages: stage II and stage III (p=0.03) (**Figures 5A, B**). Meanwhile cg07380026 had significant differences between N1 and N3 (p=0.04) and between T2 and T3 (p=0.02) (**Figures 5C, D**). The CpG site cg26006951 still shows no correlation with clinicopathological features in the TCGA cohort. This result is consistent with that of SYSUCC cohort.

### TERT gene expression and DMSs were correlated with immune infiltration in TNBC patients

**Figure 6:**
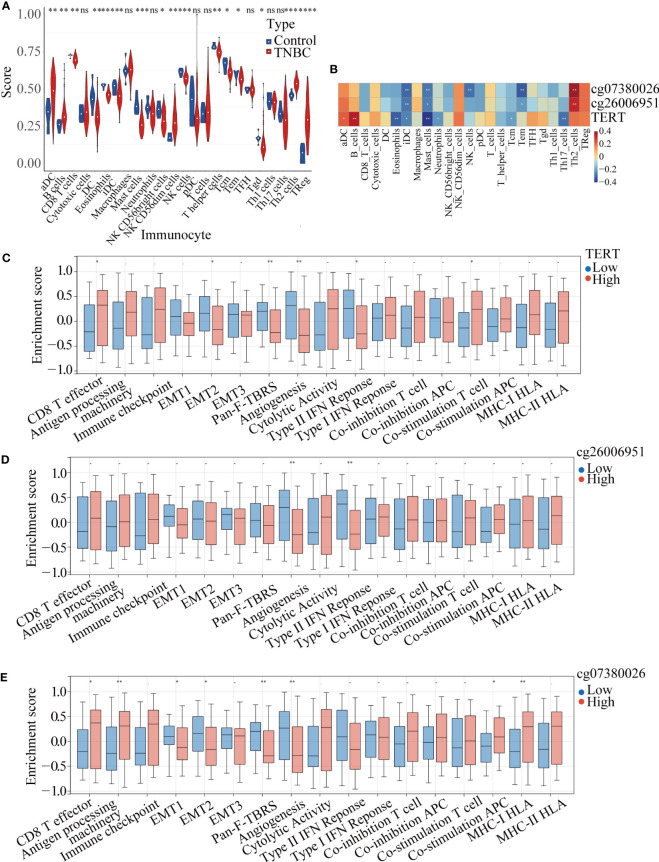
TERT expression and promoter methylation were correlated with immune cell infiltration in TNBC. **(A)**. Immune infiltration scores for tumor and normal tissues in TNBC. **(B)**. Correlation heatmap of TERT expression and two differentially methylated CpG sites (cg26006951 and cg07380026) with 24 types of immune cells in the TCGA cohort. **(C–E)**. Relative enrichment scores for 17 immune-related pathways in the high-TERT-expression and low-TERT-expression groups based on the median scores for TERT expression or cg26006951 and cg07380026 methylation level. * P < 0.05; ** P < 0.01; ns, not statistically.

ssGSEA of the samples from TNBC patients and normal samples identified significant differences in the infiltration levels of 16 immune cell types, including activated dendritic cells (aDCs), B cells, CD8^+^ T cells, and DCs, indicating that the immune microenvironment played a crucial role in TNBC (**Figure 6A**). We further analyzed the correlation among TERT expression, DMSs, and 24 types of immune cells, and found that high TERT expression and hypermethylated CpG sites within the TERT promoter (cg07380026 and cg26006951) were associated with more extensive immune infiltration of aDCs, B cells, cytotoxic cells, CD56^dim^ NK cells, T cells, T helper 2 (Th2) cells, and regulatory T cells (Tregs). We also showed that low TERT expression and hypomethylation of CpG sites within the TERT promoter (cg07380026 and cg26006951) were associated with increased immune infiltration of eosinophils, iDCs, mast cells, neutrophils, CD56^bright^ NK cells, NK cells, T helper cells, central memory T cells, effector memory T cells, Th1 cells, and Th17 cells. Among the 24 immune cells examined, the increases in iDC and mast cell infiltration levels were statistically significant (**Figure 6B**). Moreover, we divided the TNBC patients into high-risk and low-risk groups according to the median value of TERT expression and the beta-value of TERT DMSs (cg07380026 and cg26006951) and then quantified 17 immune-related biological pathways in TNBC patients from TCGA using the R package “gsva” (version 1.40.1). The results showed that high TERT expression was correlated with lower epithelial mesenchymal transition 2 (EMT 2), pan-fibroblast TGFβ response signature (Pan-F-TBRS), angiogenesis, and type II interferon (IFN) response scores. Additionally, high TERT expression was associated with higher CD8^+^ T effector scores and higher co-stimulation T-cell scores, indicative of abundant immune cell infiltration in the tumor microenvironment (**Figure 6C**).

Furthermore, we also analyzed the correlation of the cg07380026 and cg26006951 methylation status with 17 immune-related pathways. We found that cg07380026 hypomethylation was associated with lower CD8+ T effector, antigen processing machinery, APC co-stimulation and MHC-I HLA scores and with higher EMT1, EMT2, Pan-F-TBRS and angiogenesis scores (**Figures 6D, E**). Moreover, cg26006951 hypomethylation was associated with higher angiogenesis and type II IFN response scores. These results suggest that TERT, cg07380026, and cg26006951 exerted similar effects on angiogenesis, implying that patients with elevated TERT expression and cg07380026 and cg26006951 hypermethylation were likely to be insensitive to anti-angiogenesis therapy.

### TERT gene expression and DMSs were correlated with key immunomodulators in TNBC patients

**Figure 7:**
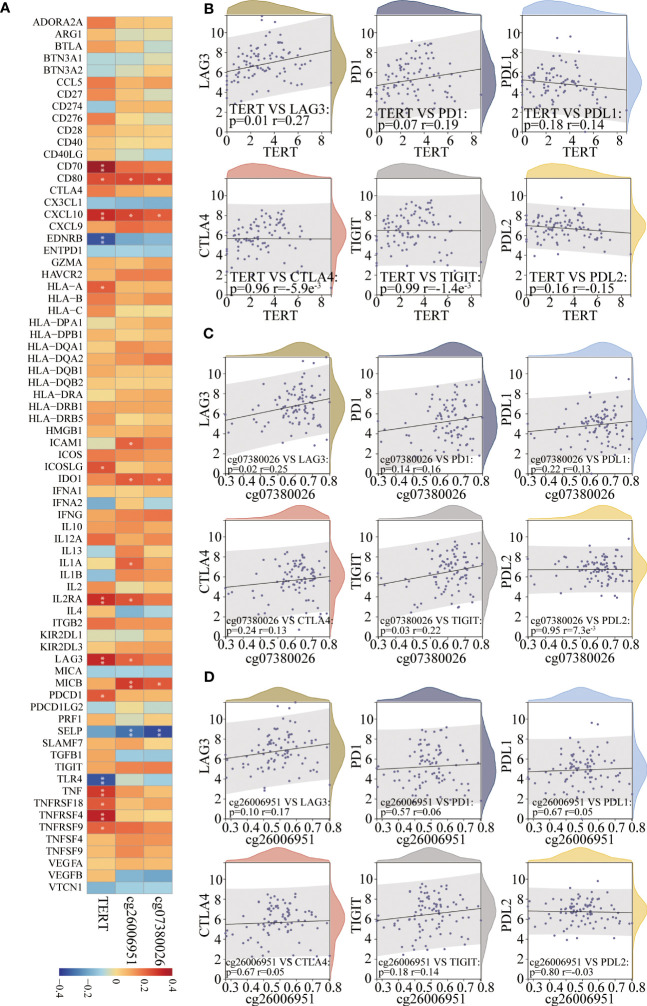
TERT expression and promoter CpGs were associated with the expression of key immunomodulators in TNBC. **(A)**. Correlation heatmap of TERT expression and two differentially methylated CpG sites (cg26006951 and cg07380026) with 74 key immunomodulators in the TCGA cohort. **(B–D)**. The correlation of TERT expression, cg26006951, and cg07380026 methylation level with the immune checkpoint molecules LAG-3, PD-1, PD-L1, CTLA4, TIGIT, or PD-L2, respectively. * P < 0.05; ** P < 0.01.

Spearman’s algorithm was adopted to analyze the correlations among TERT gene expression, TERT DMSs, and 74 immunomodulators. Significant differences were found for 27 immunomodulators (**Figure 7A**; **Supplementary Table 3**). We also analyzed the correlations among TERT expression, cg07380026, cg26006951 and routine immune checkpoint molecules (LAG-3, PD-1, PD-L1, CTLA4, TIGIT, and PD-L2), and showed that LAG-3 was significantly positively associated with TERT expression and cg07380026 and cg26006951 hypermethylation (**Figures 7B–D**).

**Figure 8:**
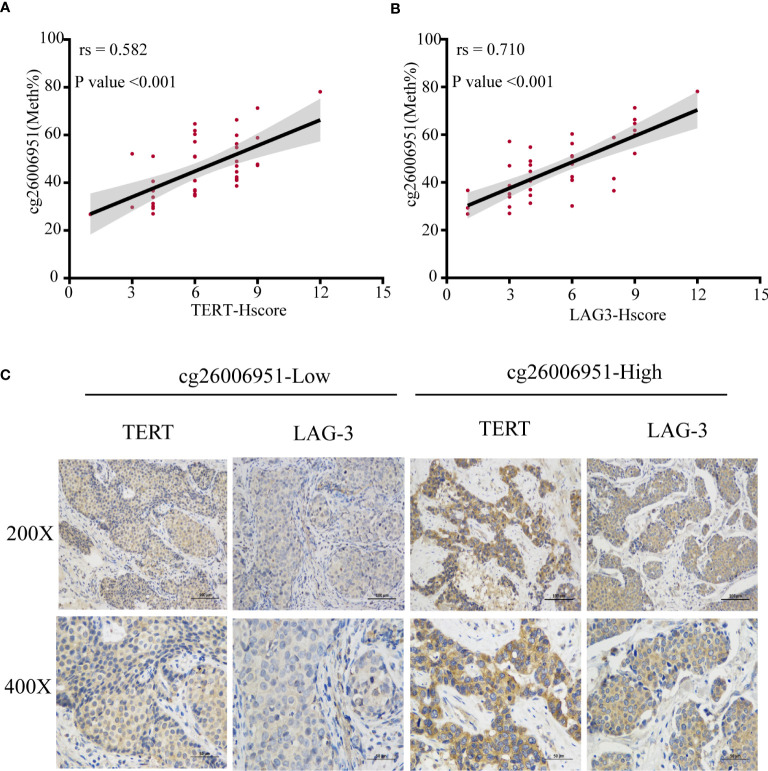
The methylation of cg26006951 is positively correlated with the expression of TERT and LAG-3. **(A, B)**. The correlation of cg26006951 methylation with LAG-3 and TERT expression in the SYSUCC cohort. **(C)** Representative immunohistochemistry images of LAG-3 and TERT expression in the cg26006951 hyper- and hypomethylation groups in the SYSUCC cohort.

To verify the association between LAG-3 and TERT promoter methylation or TERT expression, we examined the expression of LAG-3 and TERT and the methylation status of the TERT promoter in 40 TNBC tissues in the SYSUCC cohort. We found that cg26006951 hypermethylation was positively correlated with TERT and LAG-3 expression (**Figures 8A-C**). These results further validated that TERT promoter methylation was associated with overall survival and immunomodulator-LAG-3 in TNBC patients.

## Discussion

Epigenetics refers to a gene expression regulatory mechanism that affects transcription and translation without altering the DNA sequence (46, 47). Although genetic factors also underlie the etiology of many tumors, epigenetic abnormalities lead to more in-depth and extensive perturbations of cell signaling pathways and are thus more conducive to tumor occurrence and development (48, 49). The mechanisms involved in epigenetic regulation include DNA methylation, histone modifications, chromatin remodeling, and RNA interference (50). DNA methylation refers to the covalent modification of cytosine (51). In cancer, abnormal DNA methylation usually comprises hypermethylation of the promoters of suppressor genes and hypomethylation of those of oncogenes (52). The dysregulated methylation of CpG islands in the promoter regions of suppressor genes can result in their silencing and inactivation. This affects the normal expression of the affected genes, leading to uncontrolled cell proliferation and the promotion of tumor occurrence and development (52).

Telomere maintenance, which enables tumor cells to replicate indefinitely, is an important feature of most tumors (53–55). Most cells do not display telomere maintenance mechanisms (56). However, in stem cells, germ cells, and activated memory lymphocytes, among other cell types, the telomerase complex actively maintains telomere length (22, 57). Previous studies have shown that telomerase is tightly regulated in normal cells (22, 58, 59). TERT is the key rate-limiting catalytic subunit of telomerase and most tumor cells maintain telomere length by abnormally upregulating TERT expression (22, 60).

In this study, we showed that TERT expression was elevated in patients with TNBC and four differential CpG sites in the TERT promoter were hypermethylated. Further correlation analysis revealed that three of them were significantly positively correlated with TERT expression. These findings suggest that aberrant TERT promoter hypermethylation might be responsible for the elevated TERT expression in TNBC. Similarly, different studies in other tumor types have drawn similar conclusions with ours. It has shown that the TERT promoter is hypermethylated in both tumor tissues and cancer cells and is positively correlated with TERT mRNA expression and telomerase activity (61–64). In view of the widespread abnormal hypermethylation of the TERT promoter in different tumor types, many scholars have also explored its clinical prognostic value. Pedro Castelo-Branco et al. from the University of Toronto in Canada investigated whether TERT promoter methylation can be used as a biomarker of malignancy and a marker of prognosis in pediatric brain tumor patients (65). They found that TERT promoter hypermethylation was positively correlated with TERT expression, and TERT promoter hypermethylation was associated with disease progression and poor prognosis in children with brain tumors. They also found that regarding the identified CpG site cg11625005, in 79 normal brain tissues and low-grade tumor tissues, 78 samples (99%) showed no hypermethylation, while among the 201 malignant tumor samples, 145 samples (72%) were hypermethylated, and the results were statistically significant. Meritxell Oliva et al. (66) generated array based DNAm profiles describing methylation and transcriptome correlations for 987 human samples (9 tissue types from 424 subjects) and found that hypermethylation of cg07380026 might affect TERT expression in breast and ovarian cancer. Subsequently, a series of studies have shown that hypermethylation of TERT promoter is associated with poor prognosis in tumors such as melanoma (67), gastric cancer (68), bladder cancer (69), prostate cancer (70) and thyroid cancer (71). Although studies mentioned above have shown that TERT promoter hypermethylation is positively correlated with TERT expression, some studies have obtained contradictory results. One study has concluded that the causal relation between TERT promoter methylation and gene expression remains to be established (72). What’s more, in another liver cancer study, Iliopoulos et al. showed that there was a significant negative correlation between TERT expression and TERT promoter methylation (73). The reason for the opposite conclusion may be that TERT promoter methylation detection regions selected in different studies are different because each region contains different CpG methylation sites, and the methods of detecting methylation are also different in different studies. In view of the above reasons, there may be some differences in the results of different studies, and even some conclusions are controversial.

In the present study, we also sought to identify immune biomarkers related to high TERT expression and TERT promoter hypermethylation. We found that LAG-3, an immune checkpoint receptor protein, was positively correlated with high TERT expression and TERT promoter hypermethylation. The primary function of LAG-3 involves the negative regulation of T-cell function, the maintenance of immune system homeostasis, and the promotion of tumor immune escape (74). LAG-3 shows great potential as a target in tumor immunotherapy (75). Additionally, LAG-3 may be a better immunotherapeutic target than PD-1 or CTLA-4 given that, although antibodies against the PD-1 and CTLA-4 immune checkpoints can activate effector T cells, they cannot inhibit Treg activity (76). Studies have shown that a LAG-3 antibody can activate T effector cells while inhibiting Treg activity (76). Like PD-1 and CTLA-4, LAG-3 is not expressed on naive T cells but can be induced on CD4^+^ and CD8^+^ T cells upon antigenic stimulation (77). LAG-3 is also expressed in subsets of CD4^+^ T cells with suppressive function. Foxp3^+^ Tregs constitutively express LAG-3 (78). Taken together, our study demonstrated that TERT promoter hypermethylation was not only associated with TERT expression and overall survival in TNBC but also significantly positively correlated with expression of immune checkpoint molecules–LAG-3. It is intriguing to continue to investigate whether TERT is capable of regulating the expression of LAG-3 directly. If so, the combination of TERT inhibitors and LAG-3 inhibitors in solid tumors is a promising strategery.

Although we reported this important finding for the first time, this study had some limitations. Because patients with TNBC account for approximately only 15% of all breast cancer patients, the sample size in this study (40 TNBC patients) was relatively small and the validation cohort was derived from only one single cancer center (SYSUCC). We searched for other databases that could serve as external validation cohorts, such as CPTAC. However, we could only find five samples from TNBC patients. Considering that the sample size was too small, we excluded these five samples from the analysis. Furthermore, we only used immunohistochemical methods to indirectly demonstrate that high TERT expression was positively correlated with high LAG-3 expression at the protein level. Additionally, we did not elucidate the mechanism underlying the identified correlation between the high TERT and LAG-3 expression levels. These limitations provide a direction and reference for our future research.

## Data availability statement

The original contributions presented in the study are included in the article/**Supplementary Material**. Further inquiries can be directed to the corresponding authors.

## Ethics statement

The clinical specimens used in this study were obtained from the Tumor Biobank of SYSUCC. The study protocol for the SYSUCC cohort was approved by the Institutional Research Ethics Committee of SYSUCC (B2022-332-01).

## Author contributions

FL, JH, LG, WD and HL conceived of the study and participated in its design and coordination. FL, JH, TJ, WZ, WX, JG and MC performed the experiments and statistical analysis. FL, WD and MC drafted and revised the manuscript. All authors read and approved the final manuscript.
